# Derivation and 97% Purification of Human Thyroid Cells From Dermal Fibroblasts

**DOI:** 10.3389/fendo.2020.00446

**Published:** 2020-07-15

**Authors:** Risheng Ma, Rui Shi, Syed A. Morshed, Rauf Latif, Terry F. Davies

**Affiliations:** Thyroid Research Unit, Department of Medicine, Icahn School of Medicine at Mount Sinai and James J. Peters VA Medical Center, New York, NY, United States

**Keywords:** induced pluripotent stem cells (iPSCs), thyroid differentiation, PAX8, NKX2-1, TAZ, NIS, TSH receptor (TSHR)

## Abstract

**Background:** The success in rescuing thyroid deficiency in mice using thyroid cells derived from embryonic stem (ES) cells, together with the discovery of human induced pluripotent stem cells (iPSCs) from somatic cells, has raised the possibility of patient-specific thyroid cell replacement. In this study we demonstrate that human thyroid follicular cells can be derived from human iPSCs and show the ability of highly purified and differentiated cells to secrete thyroid hormone.

**Research Design and Methods:** Human iPSCs were derived from adult skin fibroblasts using RNA reprogramming and differentiated *in vitro* into thyroid follicular cells by exposure to activin A, ethacridine and TSH as we have previously described for human ES cells. The resulting thyroid cells were then highly purified using double antibody cell sorting.

**Results:** The iPSCs derived from human dermal fibroblasts showed stem cell-like morphologic changes and expressed pluripotent stem cell markers as assessed using qPCR, immunofluorescence staining, and FACS analysis. These cells retained their pluripotential characteristics as shown by teratoma formation after murine transplantation. Definitive endoderm cells were induced with activin A and the transcription factor TAZ was significantly induced on ethacridine treatment and translocated to the nucleus. Thyroid transcription factors NKX2-1 and PAX8 were also highly expressed in activin A derived endoderm cells and further induced by ethacridine. Following terminal differentiation with TSH, there was enhanced thyroid follicle formation, high expression of the thyroid specific genes—TG, TPO, TSHR and NIS, and secretion of thyroid hormone (T4) *in vitro*. Furthermore, we were able to achieve a 97% purification of TSHR+/NIS+ expressing cells after differentiation using a single purification procedure.

**Conclusions:** These findings demonstrate that mature adult dermal fibroblasts can be matured into human iPSCs which have the potential to form functional thyroid follicular cells. This lays the groundwork for future person–specific thyroid regenerative therapy.

## Introduction

Thyroid deficiency, as an irreversible pathological change, occurs due to congenital abnormalities, autoimmune thyroiditis, radioiodine ablation therapy and of course after thyroid surgery. Embryonic stem (ES) cells hold great promise for providing an unlimited source of thyrocytes since ES cells self-renew indefinitely in cell culture (1) and can be differentiated into functional thyrocyte-like cells (2, 3). iPS technology allows the generation of pluripotent stem cells from any somatic cell and offers the potential of autologous thyroid cell transplantation since patient-specific cells can be used for cellular reprogramming (4). It is, therefore, predicted that patient specific iPS cells will become not only powerful tools for biological research but also a potent source for regenerative medicine procedures (5).

In the current study, we have differentiated thyroid follicular cells from integration-free and virus-free iPSCs reprogramed from human skin fibroblasts using a single transfection step of self-replicating synthetic RNA for *OCT4, KLF4, SOX2, and GLIS1*. A protocol we have previously used to generate thyroid cells from embryonic stem cells (2) was then used to prepare functional thyroid follicular cells. Using mRNA detection via qPCR and using protein detection via immunofluorescence, we show that such terminally differentiated follicular cells have the potential to assemble into thyroid follicles and secrete thyroid hormone which opens the road to thyroid cell replacement therapy for selected patients with thyroid deficiency.

## Materials and Methods

### Ethical Statement

This study was approved by the medical research ethics committee of The Icahn School of Medicine at Mount Sinai. Informed consent was obtained from the volunteer subjected to a skin biopsy.

### Proliferative Culture of Human Skin Fibroblasts

A dermal skin core biopsy was used to generate a human skin fibroblast culture as described previously (6). A freshly isolated skin sample taken from a 59 year old healthy male was dissected and placed in 0.1% gelatin coated 6-well plates containing 1 mL of DMEM High-Glucose Medium (EMD Millipore) with 10% fetal bovine serum (EMD Millipore, Burlington, MA) with 1% GlutaMAX (Life technologies Corporation, Grand Island, NY) at 37°C with 5.0% CO_2_. The media was changed every 2–3 days and once the fibroblasts were 70–90% confluent the cells were passaged using 0.25% trypsin (Thermo Fisher Scientific, Waltham, MA) and re-plated onto 0.1% gelatin (Sigma-Aldrich, St. Louis, MO) coated plates (passage #1). The tissue pieces did not attach and were washed out by the media change.

### Reprogramming of Human Skin Fibroblasts to iPS Cells

Reprogramming was performed using the Simplicon™ RNA Reprogramming Kit (OKSG) (EMD Millipore, Burlington, MA) on the human skin fibroblast cells at passage #2. One day before reprogramming, 2 × 10^5^ cells were plated in a 6-well plate (Corning, Corning, NY) coated with 2% Matrigel (Sigma-Aldrich, St. Louis, MO) in culture media in order to have the cells reach 60–80% confluency the next day. On the day of reprogramming, the fibroblasts were pre-treated with Advanced DMEM (Life Technologies Corporation, Grand Island, NY) containing 200 ng/ml Human Recombinant B18R protein (EMD Millipore, Burlington, MA) for 2 h to help suppress the cellular interferon response, then cells were transfected once with a total of 1 μg RNA mixture (0.5 μg VEE-OKS-iG/0.5 μg B18R mRNA) was transfected with Lipofectamine 2000 in 1 ml opti-MEM (Life Technologies Corporation, Grand Island, NY). After 3 h, the transfection medium was changed to the Stage 1 media: Advanced DMEM containing 200 ng/mL of B18R protein, 2 mM L-glutamine (Life Technologies Corporation, Grand Island, NY) and 10% FBS (GE Healthcare, Chicago, IL). Puromycin (Invivogen, San Diego, CA), 0.1 μg/mL, was then added to Stage 1 media after daily media changes until day 9 post-transfection. Ten days after transfection, cells were detached using Accumax™ (Sigma-Aldrich, St. Louis, MO) and 1.0 × 10^5^ transfected cells were plated in one well of a 6-well plate (Corning, Corning, NY) in Stage 2 media which consisted of MEF conditioned medium (R & D Systems, Minneapolis, MN) supplemented with 10 ng/mL bFGF (R&D systems, Minneapolis, MN), TGF-β RI Kinase Inhibitor IV, Sodium Butyrate and PS48 (all from Simplicon) and 200 ng/mL B18R protein (EMD Millipore, Burlington, MA). The Stage 2 media was changed every other day. Small colonies appeared on day 14 after transfection and by day 18, B18R protein was removed from the media. Putative human iPS cell (hiPSc) colonies were manually picked on day 21 and re-plated in 2% Matrigel coated plates in regular human ES cell media (mTeSR1 or StemFlex) and expanded in iPSc media at 37°C in a 5% CO2 atmosphere. This line of hiPS cells is subsequently referred to as LF cells.

### Teratoma Formation

The procedure for assessing teratoma formation in nude mice was approved by the Animal Care and Use Committee of the Icahn School of Medicine at Mount Sinai. Undifferentiated hiPSCs (1 × 10^6^ cells) suspended with 100 μl of Matrigel (BD Biosciences, Franklin Lakes, NJ) were injected subcutaneously into 6–8 week-old Immunodeficient nude mice (Jackson Laboratories, Farmington, CT USA). Four weeks after injection, the formed teratomas were fixed with 10% formalin and processed for hematoxylin & eosin staining and histological evaluation.

### Differentiation of Human iPS Cells

**Figure 1:**
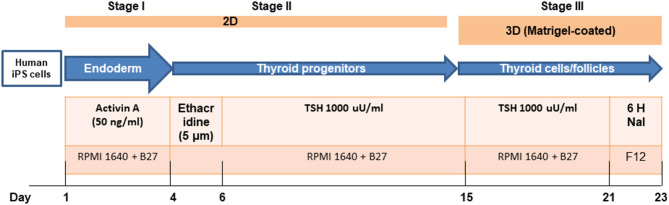
A sequential treatment scheme for differentiation of thyroid cells from human iPSCs (LF cells).

For thyroid cell differentiation, 1 × 10^6^ of HiPSCs were plated into 6-well plate and cultured for 2 days. To initiate endoderm differentiation HiPSCs were washed once with PBS and cultured in RMPI 1640 medium (Life Technologies Corporation, Grand Island, NY) containing 1% B27 (Life Technologies Corporation, Grand Island, NY) supplemented with 100 ng/ml of activin A (R&D Systems, Minneapolis, MN) for 4 days. Subsequently, the supplements were changed to ethacridine (Sigma-Aldrich, St. Louis, MO) (concentration as indicated in each experiment) to enhance the transcriptional activity of PAX8 and NKX2-1 over 2 days. This was followed by further exposure of these cells to bovine TSH (1 mU/ml) for the induction of thyroid differentiation for up to 21 days in all (Figure 1). Then, the cells were embedded in growth factor-restricted Matrigel (BD Biosciences, Franklin Lakes, NJ) and placed into 6-well plates in RPMI 1640 containing 1% B27 (LifeTechnologies Corporation, Grand Island, NY) supplemented with 1 mU/ml TSH to form thyroid follicles.

### Gene Expression Analysis

For gene expression analysis, total RNA was extracted using an RNeasy kit (Qiagen Ltd, Manchester, UK) and was treated with ribonuclease-free deoxyribonuclease (Qiagen Ltd, Manchester, UK). Five micrograms of total RNA were reverse transcribed into cDNA using the SuperScript III system (Invitrogen, San Diego, CA). Real-time quantitative RT-PCR (qRT-PCR) was carried out using a SYBR green qPCR master mix (Applied Biosystems, Foster City, CA) employing the StepOnePlus system (Applied Biosystems, Foster City, CA). Relative expression levels of each gene in real-time were analyzed using the 2^−−ΔΔCT^ method and normalized to the expression of the housekeeping gene GAPDH. Data presented are from three independent experiments in which all sample sets were analyzed in triplicate.

### Immunofluorescence Staining

Cells were grown in Delta T culture dishes and fixed with 4% paraformaldehyde in phosphate-buffered saline (PBS) and rinsed three times with PBS. After being permeabilized in methanol at −20°C for 10 min, the cells were blocked for 60 min with 5% normal goat serum in PBS and then incubated with appropriate primary antibodies (for details see Table 1) in 0.1% Tween 20 and 1% BSA in PBS overnight at 4°C. Cells were rinsed three times with PBS and incubated with the appropriate secondary antibody and then washed twice with PBS, and mounted using hard set mounting media containing DAPI (Vector Laboratories, Burlingame, CA). Fluorescence micrographs were acquired using an LSM 700 confocal microscope with a 63X objective and images were processed and assembled in Photoshop CS (Adobe, San Jose, CA, USA).

**Table 1:** Antibodies used in the described Methods.

	**Name**	**Host**	**Source**	**Catalog #**	**~Dilution**	
					**Immuno fluorescence**	**Flow Cytometry**
**Primary antibodies**	Polyclonal to OCT4 antibody	Rabbit	Cell Signaling Technology, Danvers, MA	2,750	1:200	1:200
	mAb to SOX2	Rabbit	Cell Signaling Technology, Danvers, MA	3,579	1:400	1:300
	mAb to TRA1-81	Mouse	Cell Signaling Technology, Danvers, MA	4,745	1:1000	1:1500
	Polyclonal to NANOG antibody	Rabbit	Cell Signaling Technology, Danvers, MA	3,580	1:800	1:400
	Monoclonal to FOXA2 antibody	Mouse	Thermo Fisher Scientific, Waltham, MA	MA5-15,542	1:500	1:500
	Monoclonal to SOX17 antibody	Rabbit	Cell Signaling Technology, Danvers, MA	81,778	1:1000	1:1000
	Monoclonal to NKX2-1 antibody	Rabbit	Abcam, Cambridge, UK	ab76013	1:400	1:500
	Monoclonal to PAX8 antibody	Mouse	Thermo Fisher Scientific, Waltham, MA	MA1-117	1:100	1:100
	Monoclonal to TAZ antibody	Rabbit	Cell Signaling Technology, Danvers, MA	8,418	1:100	–
	Monoclonal to TG antibody	Rabbit	Abcam, Cambridge, UK	ab156008	1:200	–
	Monoclonal to NIS antibody	Mouse	Thermo Fisher Scientific, Waltham, MA	MA5-12,308	1:500	–
	Polyclonal NIS	Rabbit	Self-generated by Genscript, Piscataway, NJ.		–	0.2 ug IgG/10^6^ cells
	mAb TSHR	Mouse	RSR Ltd, Cardiff, UK	RSR-4	–	1 ug IgG/10^6^ cells
**Polyclonal secondary antibodies**	Anti-rabbit IgG (H+L), F(ab')2 Fragment (Alexa Fluor^®^ 488 Conjugate)	Goat	Cell Signaling Technology, Danvers, MA	4,412	1:1000	1:1000
	Anti-mouse IgG (H+L), F(ab')2 Fragment (Alexa Fluor^®^ 488 Conjugate)	Goat	Cell Signaling Technology, Danvers, MA	4,408	1:1000	1:1000
	Anti-rabbit IgG (H+L), F(ab')2 Fragment (Alexa Fluor^®^ 555 Conjugate)	Goat	Cell Signaling Technology, Danvers, MA	4,413	1:1000	1:1000
	Anti-mouse IgG (H+L), F(ab')2 Fragment (Alexa Fluor^®^ 555 Conjugate)	Goat	Cell Signaling Technology, Danvers, MA	4,409	1:1000	1:1000
	Anti-rabbit IgG (H+L), F(ab')2 Fragment (Alexa Fluor^®^ 647 Conjugate)	Goat	Cell Signaling Technology, Danvers, MA	4,414	1:1000	1:1000

### Thyroxine (T4) Measurement

After 21 days, the differentiated human iPSCs were cultured in modified Ham's F12 medium (Thermo Fisher Scientific, Waltham, MA) supplemented with 5% bovine calf serum (Sigma-Aldrich, St. Louis, MO) and seven hormones including bovine TSH (1 U/L), insulin (10 mg/L), hydrocortisone (0.4 mg/L), human transferrin (5 mg/L), glycyl-l-histidyl-l-lysine acetate (10 μg/L), somatostatin (10 μg/L), and NaI (10 ug/L). After 48 h, the spent culture supernatant was harvested for measurement of T_4_ using the Millipore MAP thyroid magnetic bead method (Cat # RTHYMAG-30 K) as per the manufacturer's protocol. The undifferentiated LF-cells grown in the same conditions were used as negative controls. The Fisher rat thyroid cells (FRTL-5) secreted T4 when grown in the six hormones medium (7) and were used as a positive control. All the chemical reagents were purchased from Sigma-Aldrich unless specified. All cells were cultured in Matrigel–coated dishes.

### Purification of Double Positive Cells

Since NIS and TSHR are two thyroid specific surface markers that are expressed during differentiation we used these markers to purify the cells using flowcytometry. In brief, confluent layers of differentiated cells were harvested with 1 mM EDTA/EGTA in PBS (calcium- and magnesium-free). After washing once with 1XPBS, 1 × 10^7^ cells were incubated for 30 min at 4°C with monoclonal anti-TSHR (RSR4) directed to the TSHR epitope aa 322–341- at 1 ug/10^6^cell and a rabbit polyclonal anti-human NIS antibody at 0.2 ug/10^6^cells (Table 1) with intermittent shaking. A new NIS polyclonal antibody to a 15 mer external epitope (MEAVETGERPTFGAC) in the extracellular portion of NIS was used after characterization with FRTL-5 cells (see Supplementary Figure 1). After two washes with sterile PBS, the cells were labeled with anti-mouse IgG conjugated with Alexa Fluor 647 and anti-rabbit IgG conjugated with Alexa Fluor 488 diluted to 1:1000 in RPMI medium containing 1 mM EDTA/1 mM EGTA plus 1% BSA for 20 min at 4°C, then washed twice and resuspended in complete medium. Cells stained by a similar protocol with anti-mouse IgG conjugated to Alexa Fluor 647 and rabbit IgG conjugated to Alexa Fluor 488 were used as controls to draw the gates for positive and negative stained cells. The stained cell suspension was then sorted using a BD FACSAria™ III sorter and analyzed using BD DIVA software. The degree of purification of the sorted cells was ascertained by checking the FACS before and after purification. Undifferentiated cells were used as negative control to show the specificity of staining.

## Results

### Generation and Characterization of Human Induced Pluripotent Stem Cells (iPSCs)

**Figure 2:**
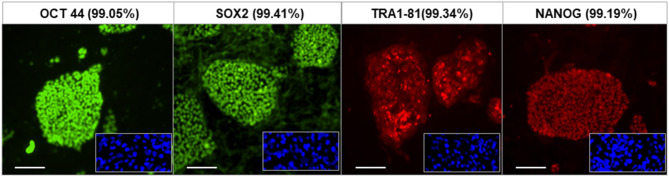
Characterization of human iPSCs (LF cells). Expression of the pluripotent markers OCT4, SOX2, TRA1-81, and NANOG in human iPS cells detected by immunofluorescence and FACS (date shown as % in bracket). Insets show control fibroblasts. Scale = 100 μm.

The fibroblasts from human skin initially appeared spindle-shaped. After transfection and reprogramming the cells showed epithelioid changes and mature iPSCs (named LF cells) had regular circular shape in a compact colony, resembling that of human ES cells, and formed typical distinct embryoid bodies (not shown). We confirmed the pluripotency of derived human iPSCs by their expression of cell markers including OCT4, TRA-1-60, NANOG, and SSEA4 and by FACS analysis (Figure 2). *In vivo*, teratomas were formed in nude mice injected with hiPSCs (LF cells). Three germ layers formed structures in the teratomas as confirmed by histological analysis, indicating the pluripotency of these cells (data not shown).

### Induction of Endoderm From Human iPSCs

**Figure 3:**
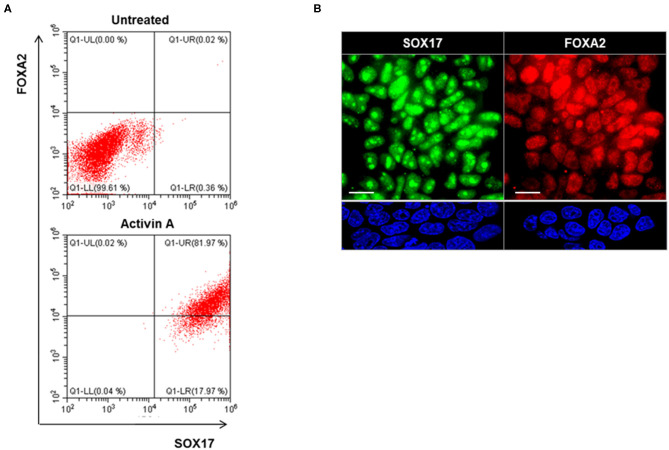
Endoderm induction in human iPSCs (LF cells). **(A)** Representative flow cytometry of SOX17 and FOXA2 expressing cells in activin A treated (lower) and untreated (upper) cells. Data were representative of three separate experiments on LF cells treated with activin A for 4 days. **(B)** Representative images of immunostaining for endoderm markers, SOX17 (Green), and FOXA2 (Red), in in activin A treated LF cells for 4 days (upper panel) and untreated cells (lower panel). Scale bar = 20 μm.

**Figure 4:**
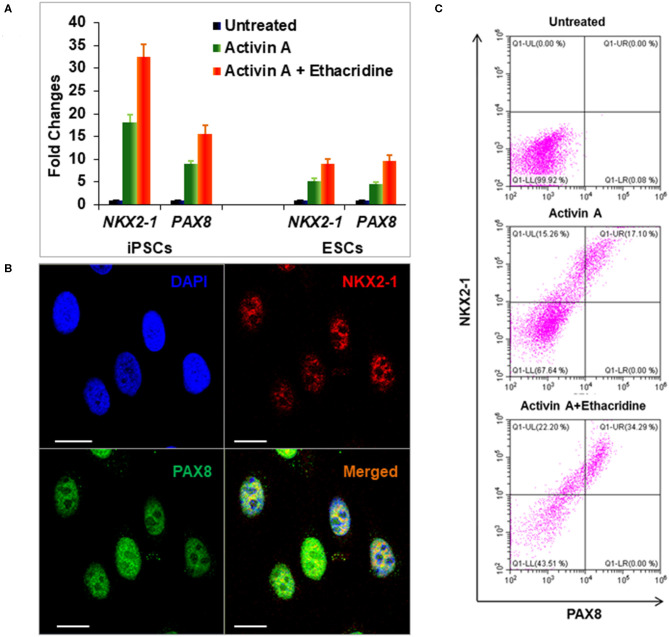
Differentiation of definitive thyroid endoderm from human iPSCs (LF cells). **(A)** qPCR analysis of thyroid transcriptional factors NKX2-1 and PAX8 in human ES cells and human iPSCs (LF cells). Fold change is represented as the mean ± SEM of three independent experiments. **(B)** Immunostaining of NKX2-1 (Red) and PAX8 (Green) in human iPSCs (LF cells) derived thyroid endoderm cells. Scale bar = 20 μm. **(C)** Representative flow cytometry of NKX2-1 and PAX8 expressing cells in untreated (upper), activin A treated (middle) and activing A + Ethacridine (lower) cells. Data were representative of three separate experiments.

Definitive endoderm cells were first induced by exposure of human iPSCs to activin A for 4 days as evidenced by their expression of the endoderm markers SOX17 and FOXA2 which were significantly induced and highly expressed in activin A treated cells in comparison to control iPSCs when assessed for protein expression by flow cytometry immunostaining (Figure 3A) and immunostaining flow cytometric analysis (Figure 3B). These activin A derived endoderm cells also expressed high levels of the transcription factors NKX2-1 and PAX8 in comparison to the human ES cells as detected by qPCR (Figure 4A), immunostaining (Figure 4B) and FACS analysis (Figure 4C) as previously reported (2). The results showed that human endodermal cells were successfully induced by activin A.

### Ethacridine as an Enhancer of Specific Transcription Factors in Human iPSC Derived Endodermal Cells

**Figure 5:**
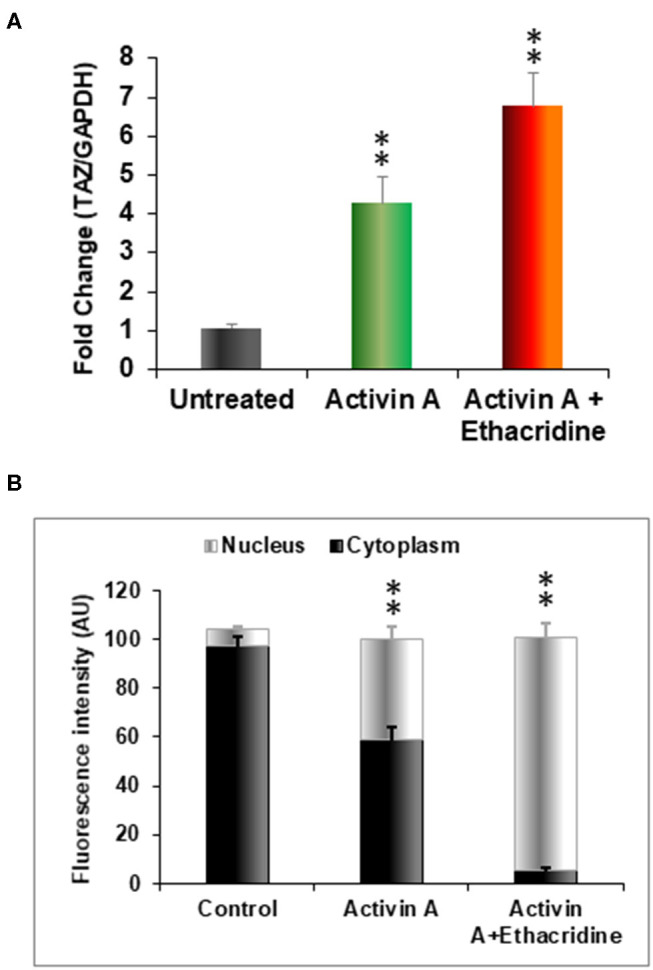
Effects of ethacridine on TAZ in human iPSCs (LF cells). **(A)** Enhancement of TAZ in ethacridine treated activin A derived endoderm from human iPSCs (LF cells). Data were expressed as mean ± SEM and represent one of three separate experiments. **p < 0.01 ethacridine treated activin A derived endoderm in comparison with untreated or activin A treated alone. Data were analyzed by analysis of variance (ANOVA) followed by the Student-Newman-Keuls test. **(B)** The relative abundances of TAZ in cells in cytoplasm and nucleus was quantified from sets of 10 cells per image and expressed as arbitrary units of Fluorescence Intensity (FI). Data are means ± SEM of three independent experiments. **P < 0.01 comparison among different groups. Data were analyzed by analysis of variance (ANOVA) followed by the Student-Newman-Keuls test.

We previously used the small molecule ethacridine to enhance speciation of human ES cells (2). We first assessed the toxicity of ethacridine on human iPSC using a cell viability/ proliferation assay after exposure to increasing doses of ethacridine for 48 h. There was no cytotoxicity of the human iPSCs up to 8 uM whereas 10 uM showed significant cell death **(data not shown)**. Hence, 5 uM ethacridine was the optimal dose that we used for thyroid cell differentiation in the subsequent studies. As expected, ethacridine significantly enhanced the mRNA expression of TAZ in comparison to untreated and activin A treated human iPSCs (*p* < 0.01) (Figure 5A). We also observed total nuclear translocation of TAZ protein in hiPSC derived human endoderm cells treated with activin A and ethacridine as detected by **quantitative fluorescence intensity** (Figure 5B) in comparison with untreated and activin A alone. Ethacridine further enhanced NKX2-1 and PAX8 in activin A derived human iPSCs (Figures 4A,C) as previously observed in human ES cells (2).

### Thyroid Cell Differentiation From Human iPSCs

**Figure 6:**
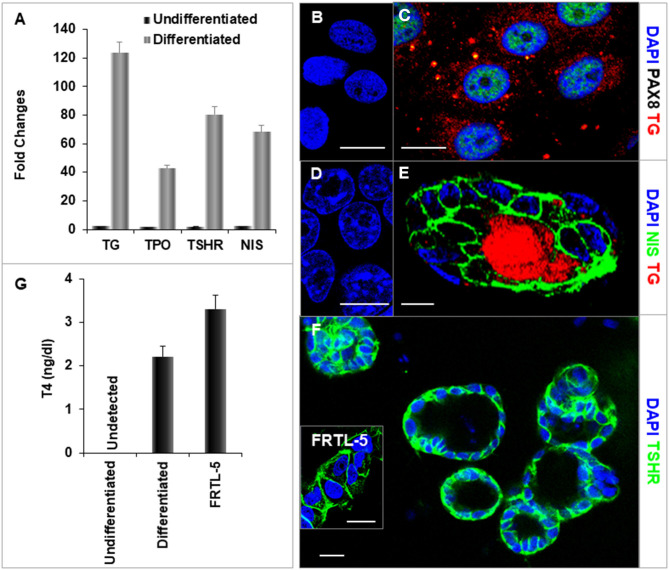
Characterization of differentiated thyroid cells. **(A)** qPCR analysis of thyroid specific genes: TG, TPO, TSHR, and NIS. Fold change is represented as the mean ± SEM of three independent experiments on differentiated LF cells at 21 days. **(B–F)** Immunostaining of thyroid genes in undifferentiated and differentiated LF cells at 21 days culture: **(B,D)** Undifferentiated cells as a control. **(C)** Staining of TG (Red) and PAX8 (Green) in differentiated cells. TG expressed in cytoplasm and PAX8 expressed in nucleus. **(E)** Staining of TG (Red) and NIS (Green) in a differentiated thyroid follicle: NIS was expressed in the membrane and TG was expressed in the cytoplasm and follicular lumen. **(F)** Thyroid neo-follicles derived from differentiated cells expressing TSHR (Green) in the membrane. Inset shows staining of rat FRTL-5 thyroid cells. Scale bar = 20 μm. **(G)** Measurement of T4 from the differentiated LF cells: T4 was detected in the iodine supplemented medium of the differentiated LF cells at 23 days culture as described in Methods and was absent in the medium of the undifferentiated cells but less than in FRTL-5 cells with 7H medium.

After further differentiation of the ethacridine treated endodermal cells with TSH, the thyroid specific genes, NIS, TSHR, Tg, and TPO each demonstrated marked expression in comparison to the undifferentiated iPSCs (Figures 6A–F). Immunostaining of TG (red) and PAX8 (green) (Figure 6C), and NIS (green) (Figure 6E) were detected in the differentiated cells and not in undifferentiated cells (Figures 6B,D). These differentiated cells formed three-dimensional thyroid follicles shown expressing the TSHR (Figure 6F) indicating their commitment to a thyrocyte fate and secreted thyroid hormone (T_4_) into their culture medium when provided with iodide (Figure 6G).

### Purification of Human Thyroid Cells

**Figure 7:**
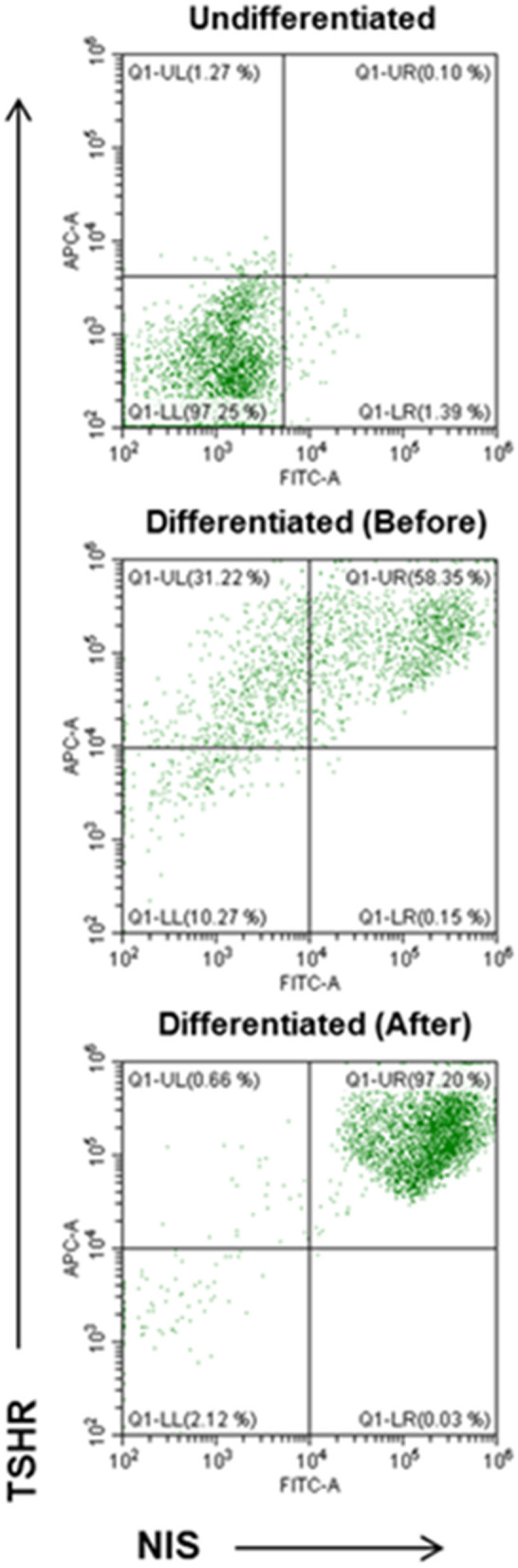
FACS analysis of TSHR and NIS expression in differentiated thyroid cells. Undifferentiated LF cells (upper), differentiated LF cells at 21 days (middle) (58.35% double positive), and purified differentiated LF cells (lower) (97.20% positive) analyzed for double anti-NIS and ant-TSHR staining after sorting purification.

Using double staining with TSHR-Ab and NIS-Ab it was possible to achieve significant purification and quantitation of the derived human thyroid cells (Figure 7). TSHR and NIS double positive cells were significantly increased from a background of 0.1% in undifferentiated LF cells to 58% in differentiated cell cultures and up to 97% cells by FACS purification.

## Discussion

Thyroid follicular cells are long lasting and have a slow turnover time of ~10 years (8). However, like other tissues, the thyroid is also subject to autoimmune destruction and cancer mutations causing cell loss or requiring gland removal. Thyroid cell replacement therapy is one approach to correct such deficiency. Patient derived IPSCs are an ideal way to make personal cell replacement possible. In this report we demonstrate the successful derivation of human iPSCs from dermal fibroblasts and the reprogramming of these cells into functional human thyroid follicular cells. Our findings provide new insight into the potential for patient-specific thyroid cell replacement therapy for the treatment of selected patients with thyroid deficiency. While there are many safety concerns that need to be thoroughly addressed these studies are “proof of concept” for this approach. The concerns that need to be addressed include the lack of cell homogeneity in the cell population, the immunogenic potential of the derived cells and their neoplastic potential, each of which requires further study with *in vivo* models.

In this report, we describe our early studies with human skin fibroblasts that were first altered with a new reprogramming system which was an efficient method to generate integration free, virus-free, human iPSCs in a single transfection step. The technology is based upon a positive single-stranded RNA species derived from non-infectious (non-packaging), self-replicating Venezuelian equine encephalitis (VEE) virus (9). The RNA replicon produces a synthetic RNA containing the reprograming factors OCT4, KLF4, SOX2, and GLIS1 (OKS-iG) as a polycistronic transcript that is able to self-replicate for a limited number of cell divisions. Once iPSCs are generated, the RNA was selectively eliminated by removing the interferon-gamma (IFNg) inhibitor, B18R, from the cell culture medium. The result was transgene-free, replicon-free iPSCs. The Gli-like transcription factor Glis1 (Glis family zinc finger 1) markedly enhances the generation of iPSCs from both mouse and human fibroblasts when it is expressed together with OKS. DNA microarray analyses show that Glis1 promotes multiple pro-reprogramming pathways, including Myc, Nanog, Lin28, Wnt, Essrb, and the mesenchymal–epithelial transition (10). Hence, Glis1 effectively promotes the direct reprogramming of somatic cells during iPSC generation. Using this commercial technology we reprogrammed human skin fibroblasts and found that iPSC colonies first appeared between days 14–18, and we then picked them on day 21. The iPSCs were characterized by immunostaining for stem cell markers and morphologically they had the capacity to form three dimensional embryoid bodies as seen with human ES cell cultures. This is likely to be a much safer approach to cellular reprogramming than the use of exogenous gene transfection (11).

The subsequent derivation of differentiated thyroid cells from these hiPSCs using activin A, ethacridine and TSH produced similar results to those we have previously reported when working with human ES cells (2). However, it should be noted that it is likely that the ease of such differentiation may vary between different hiPS cell lines. Furthermore, although the action of activin A has been fully explored (12, 13) the mechanism of action of ethacridine is unclear. Although it appears to be primarily via the induction of the transcription factor TAZ which is a signal-responsive transcriptional co-regulator that promotes the induction of NKX2-1 and PAX8 (14)—the two most essential factors for thyroid cell speciation in mammalian cells and which are only expressed simultaneously in thyroid designated progenitor cells (14), other data suggests that thyroid cells can differentiate to a certain degree without TAZ expression (15). However, ethacridine has been reported to enhance the interaction between TAZ and protein phosphatases (PP1A and PP2A) via ASPP2 or α-catenin, promoting dephosphorylation of TAZ to increase the unphosphorylated TAZ in the nucleus (16). Within the nucleus, TAZ interacts with NKX2-1 and PAX8, increases the activity of their promoters, and synergistically enhances the transcriptional effects of NKX2-1 and PAX8 on the thyroid specific genes. This was shown using an inhibitory mutant TAZ which blocked its effects on the activity of the transcription factors (14, 17). TAZ depletion also significantly reduced the number of thyroid follicle cells in zebrafish, again indicating TAZ is needed for the normal differentiation of thyroid (15). Together, these observations indicate that ethacridine has the ability to enhance translocation of TAZ into the nucleus and enhance the key thyroid transcription factors NKX2-1 and PAX8 in human iPSc derived endodermal cells.

To understand the epigenetic control of thyroid cell speciation we have previously profiled chromatin accessibility by the ATAC-seq technique and examined DNA methylation and histone acetylation of the promoter regions of thyroid-related transcription factors and thyroid specific genes during hES cell differentiation (18). ATAC-seq analysis showed enhanced chromatin accessibility in the promoter regions of TAZ, NKX2-1 and PAX8 after exposure to activin A plus ethacridine in contrast to activin A alone [Ma et al. (18) Thyroid, 2020, in press]. While there were no methylation changes in the NKX2-1, PAX8 and TAZ promoters by bisulfite sequencing we showed that acetylation of histone H4 (AcH4), especially of lysine 16 (H4K16ac), was observed in the promoters when measured by immunoprecipitation (ChIP) PCR assays and which correlated with the activity and expression of NKX2-1, PAX8, NIS, TSHR, and TG genes. These results would suggest that ethacrdine-induced epigenetic changes are transmitted to subsequent generations of cells for perpetuation of the differentation programme.

The functionality of the derived thyroid cells is clearly of paramount importance. We have previously shown that such cells derived from human ES cell cultures were able, not only to form follicles, but also to take up radioactive iodine and secrete protein-bound iodine (PBI) as evidence of an intact organification program (2). In addition, we have corrected our hypothyroid TSHR-KO mouse by transplanting differentiated mouse ES cells and raising their serum T4 levels and suppressing their TSH (Latif et al., Unpublished) as previously shown by others with normal but hypothyroid mice (3, 19). In the current study we have also demonstrated that the derived human thyroid cells were able to secrete T4 when provided with iodide thus confirming their *in vitro* functionality.

The heterogeneity of stem cell-derived thyroid cells is of much concern. The development of tumor formation may be quite common with ES derived cultures because of such cell heterogeneity but has apparently been less common with iPSC reprogrammed cells. However, long term data on the *in vivo* survival of the grafted cells and the tissue they form remains sparse. Studies with mouse and human ES cells have shown that purification can be carried out effectively by knocking-in reporter genes which allow easy selection (3, 20, 21). While it would be straight forward to derive highly purified cells with transfected markers, such as labeled NKX2-1 (3), the transplantation of such altered cells is never likely to be acceptable in patients. Furthermore, even if accepted, in the long run there would almost certainly be an immune reaction to the transgene. In contrast, we have aimed here to achieve purification by using the native expression of two surface thyroid markers—NIS and the TSHR. By this approach, in the current experiments, we achieved over 90% purification of the differentiated thyroid cells from LF cells using a double antibody approach with FACS. This purified cell approach will begin to make autologous thyroid cell therapy a more realistic aim.

In summary, we describe the successful derivation of human iPSCs using an integration free, virus-free, method, and demonstrate the differentiation and purification of these cells into functional thyroid follicular cells. As before, we employed ethacridine to enhance-expression of PAX8 and NKX2-1 in activin A derived endoderm cells and then terminally differentiated them in the presence of TSH. These differentiated iPSCs formed three-dimensional thyroid follicles and expressed thyroid specific genes and proteins and secreted T4. These results indicate the potential of a simple approach for generation of patient-specific thyroid epithelial cells.

## Data Availability Statement

The raw data supporting the conclusions of this article will be made available by the authors, without undue reservation.

## Ethics Statement

The studies involving human participants were reviewed and approved by Icahn School of Medicine at Mount Sinai. The patients/participants provided their written informed consent to participate in this study.

## Author Contributions

RM conceived the work, designed and performed experiments, and wrote the manuscript. RS performed experiments. SM designed and reviewed paper. RL conceived the work, designed experiments, and wrote the manuscript. TD conceived the work, designed experiments, and wrote the manuscript. All authors reviewed, edited and approved the manuscript.

## Conflict of Interest

The authors declare that the research was conducted in the absence of any commercial or financial relationships that could be construed as a potential conflict of interest.
