# Supplementary figures and images for: Derivation and 97% Purification of Human Thyroid Cells From Dermal Fibroblasts
Source: Front Endocrinol (Lausanne). 2020 Jul 15;11:446. doi: 10.3389/fendo.2020.00446 (PMC7373738; doi:10.3389/fendo.2020.00446)

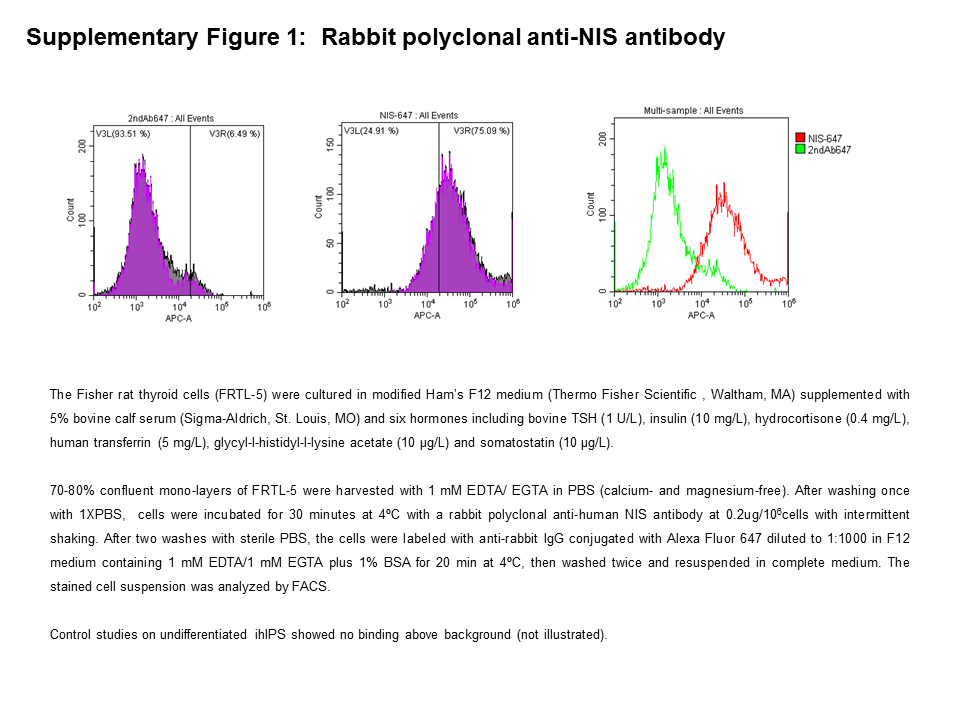

Supplement: Supplementary file 1 [file Image_1.TIF]
